# Polychlorinated Biphenyls (PCBs) Impact Prostatic Collagen Density and Bladder Volume in Young Adult Mice Exposed during in Utero and Lactational Development

**DOI:** 10.3390/toxics11070609

**Published:** 2023-07-13

**Authors:** Audrey Spiegelhoff, Kathy Wang, Monica Ridlon, Thomas Lavery, Conner L. Kennedy, Serena George, Kimberly P. Keil Stietz

**Affiliations:** Department of Comparative Biosciences, School of Veterinary Medicine, University of Wisconsin-Madison, Madison, WI 53706, USA; alspiegelhoff@medicine.wisc.edu (A.S.); kwang399@wisc.edu (K.W.); mridlon@wisc.edu (M.R.); tlavery@wisc.edu (T.L.); clkennedy3@wisc.edu (C.L.K.); sgeorge5@wisc.edu (S.G.)

**Keywords:** collagen density, persistent organic pollutants, lower urinary tract, polychlorinated biphenyls, urology, developmental basis of adult disease

## Abstract

Polychlorinated biphenyls (PCBs) are persistent organic pollutants linked to deleterious health outcomes, including voiding dysfunction in developmentally exposed mice. Changes in prostate volume and/or extracellular matrix composition are associated with voiding dysfunction in men and animal models. Whether PCB-induced changes in voiding function in male mice occur in part via alterations to the prostate or an alternate mechanism is unclear. Therefore, we tested whether developmental exposure to the MARBLES PCB mixture altered prostate morphology in young adult offspring. C57Bl/6J female mice were dosed daily with the MARBLES PCB mixture at 0, 0.1, 1 or 6 mg/kg/d for two weeks prior to mating and through gestation and lactation, offspring were collected at 6 weeks of age. Ventral prostate mass was decreased in the 1 mg/kg/d PCB group compared to other PCB groups. There were no PCB-induced changes in prostate smooth muscle thickness, apoptosis, proliferation, or testes mass. PCBs impacted the prostate extracellular matrix; anterior prostate collagen density was decreased in the 1 mg/kg/d PCB group compared to all other groups. Normalized bladder volume was increased in male and female offspring in the 6 mg/kg/d PCB group compared to control. No change in water consumption, bladder mass or bladder smooth muscle thickness accompanied changes in bladder volume. Urine and serum creatinine concentrations were elevated but only in male mice. Together, these results suggest that developmental exposure to PCBs can influence prostate wet weight and prostate/bladder morphology, but PCBs do not promote prostate enlargement. Whether these changes persist throughout adult life and how they contribute to voiding function in animal models and humans is of future interest.

## 1. Introduction

Lower urinary tract dysfunction (LUTD) can greatly affect the quality of life [1]. Common symptoms include overactive bladder, inability to empty the bladder, painful and frequent urination, and nocturia [2]. While LUTD is common in aging populations of men and women, LUTD is also prevalent in young individuals with neurodevelopmental disorders such as autism spectrum disorder (ASD) [2,3,4]. The factors which drive the development of LUTD are thought to be multifactorial, with considerable variation in age, progression, and severity. In men, a subset of LUTD can be attributed to obstruction, which can be caused by changes within the prostate. For example, benign growth of the prostate (BPH), prostatic fibrosis, and inflammation of the prostate can all lead to obstructive voiding symptoms [5,6]. A better understanding of causative agents which trigger these changes in prostatic growth or morphology could improve the ability to diagnose and treat a subset of patients suffering from LUTD. Increasing evidence suggests that early life exposure to environmental contaminants can influence prostate development, growth, and the onset and progression of abnormal voiding physiology in rodent models [7,8,9,10,11]. We have previously shown that young adult mice (6–7 weeks of age) exposed to polychlorinated biphenyls (PCBs), a ubiquitous environmental contaminant, through gestation and lactation display smaller, more frequent voiding phenotypes that were sex and dose-dependent [12]. Therefore, one goal of this study was to test whether developmental exposure to PCBs influences prostate morphology in young adult male mice, which could contribute to voiding dysfunction.

PCB congeners are broadly classified based on their structure as coplanar (dioxin-like) or non-coplanar (non-dioxin-like) [13,14]. The stability of PCBs made them desirable for use in building materials, electrical equipment, and transformers until production was banned in the US in 1979 and globally in 2001 due to health concerns [13,15]. Despite the ban, PCBs persist in the environment, in part due to legacy sources such as old building materials or waste sites [13,16]. Concerningly, contemporary sources of PCBs also exist and are produced as unintentional byproducts of several processes such as paint pigment production [17]. Due to PCB’s lipophilic nature and relative resistance to biodegradation, they bioaccumulate in organisms and biomagnify in food webs [18,19,20]. Thus, PCBs are present in food sources such as fish, livestock, and dairy milk [18,19]. Recently, PCB profiles in the serum of pregnant women in the Markers of Autisms Risk in Babies-Learning Early Signs (MARBLES) cohort include both legacy and contemporary PCBs [21,22,23]. PCBs remain a contemporary health concern, especially for pregnant women [23].

The impacts of PCBs in the lower urinary tract have only recently garnered study. It has been observed that PCBs alter bladder morphology in mice following in utero and lactational exposure, increasing bladder volume, nerve fiber density, and inflammatory cells within the bladder of juvenile 4-week-old mice in a sex- and dose-dependent manner [24,25]. Developmental PCB exposure also leads to alterations in voiding patterns in 6-week-old mouse offspring, mainly characterized as increased small spot voiding, more frequent voiding and increases in voiding pressure in a sex- and dose-dependent manner [12]. Whether PCBs influence the prostate to contribute to voiding dysfunction observed in male mice is unknown. The goals of this study are threefold. First, we aim to determine whether benign growth of the prostate coincides with previously observed PCB-induced changes in voiding function [12]. Second, regardless of changes in prostate size, we aim to determine whether PCBs alter any aspects of prostate histology in developmentally exposed offspring. Third, we aim to determine whether PCB-induced increases in bladder volume observed in 4-week-old mouse offspring [24] persist in mice that are 6 weeks of age and correlate with any changes in prostate morphology.

## 2. Materials and Methods

### 2.1. Animals

All procedures involving animals were conducted in accordance with the NIH Guide for the Care and Use of Laboratory Animals and were approved by the University of Wisconsin-Madison Animal Care and Use Committee. C57Bl/6J wild-type mice were purchased from Jackson Labs (Bar Harbor, ME, USA) and subsequently bred in-house. All mice were housed in clear plastic (polysulfone or PET) cages containing cob bedding and maintained on a twelve-hour light and dark cycle at 22 ± 2 °C. Feed (Diet 2020x, Diet 2019 (breeders), Teklad, Indianapolis, IN, USA) and water were available ad libitum. No more than 4 mice were housed per cage.

### 2.2. Developmental PCB Exposure

The MARBLES PCB mixture was used in this study as it mimics the profile of PCB congeners and abundance in the serum of a contemporary cohort of pregnant women at risk of having a child with a neurodevelopmental disorder [23]. This mix contains each of the following PCBs: 28 (48.2%), 11 (24.3%), 118 (4.9%), 101 (4.5%), 52 (4.5%), 153 (3.1%), 180 (2.8%), 149 (2.1%), 138 (1.7%), 84 (1.5%), 135 (1.3%), and 95 (1.2%). Mice were dosed with the MARBLES PCB mixture dissolved in peanut oil (Spectrum Organic Products, LLC, Melville, NY) and mixed into organic peanut butter (Trader Joe’s, Monrovia, CA) to achieve stock concentrations of 0.025, 0.25 and 1.5 mg PCB/g of peanut butter, as described previously [24,26,27]. The vehicle was peanut oil only mixed in peanut butter. Briefly, the MARBLES PCB mixture was fed daily at doses of 0, 0.1, 1, or 6 mg PCB/kg body weight/day to nulliparous female mice for two weeks prior to mating. Female mice were then paired with a male and dosed daily through mating, gestation, and lactation. The 0.1, 1, or 6 mg/kg body weight/day doses and dosing paradigm were chosen since they lead to detectable levels of PCBs in the bladder and other organs of exposed offspring at levels relevant to human health and lead to varied phenotypes in offspring [24,27,28,29]. Offspring were weaned at postnatal day (P) 21, and the group was housed with same-sex and dose littermates. Offspring were weighed and tissues were collected at 6 weeks of age (the average age of all animals used across endpoints was 45.81 ± 3.96 days old). Mice were collected from ~65 litters as part of a larger study [12] as such a subset of mice underwent anesthetized cystometry prior to euthanasia with CO2 or isoflurane overdose and exsanguination. However, bladder tissue and bladder metrics were only collected from mice that did not undergo anesthetized cystometry since the cystometry procedure makes the bladder unfit for histology.

### 2.3. Water Consumption and Bladder Metrics

Daily water consumption was measured by cage for several days starting ~1 week prior to euthanasia. Daily water consumption was then divided by the number of mice per cage and averaged across days (*n* = 4–8 cages per group, representing at least 4 litters per group). Following CO2 euthanasia, the abdomen was opened, and bladder exposed. Bladder length, width, and height were immediately measured with digital calipers (*n* = 18–28 mice per group, representing at least 7 litters per group). These measurements were then used to estimate relative bladder volume based on the volume of an ellipsoid. The term relative bladder volume is used since we cannot account for the level of distention of the bladder at the time of sacrifice, nonetheless, this measure is commonly used in conjunction with other measures to assess LUT function [7,8,9]. Bladder mass was assessed after bladders were removed and blotted on a wipe to remove excess urine (*n* = 8–21 mice per group, representing at least 4 L per group).

### 2.4. Immunohistochemistry and Tissue Staining

Following euthanasia, testes were removed and weighed, and prostate lobes were micro-dissected (anterior, dorsal, and ventral) and weighed (*n* = 13–18 mice per group, representing at least 5 litters per group). A subset of bladder and prostate tissues were fixed in 4% paraformaldehyde (Fisher, Madison, WI) overnight, and dehydrated into 100% methanol for long-term storage. Samples were rehydrated into phosphate buffered saline (PBS) (Fisher), embedded in bacto agar (VWR, Radnor, PA), and the entire block was then dehydrated into 100% ethanol, washed in xylenes (Fisher) prior to embedding in paraffin, and sectioned at 5 µm thickness. Multiple tissues from each exposure group were embedded in the same block to reduce variability in staining. Immunohistochemistry was performed as previously described [24,30,31] using antibodies and dilutions listed in Table 1. Slides were imaged with an Eclipse Ci compound microscope and DS Ri2 camera (Nikon Instruments Inc.) interfaced to NIS elements imaging software (Nikon Instruments Inc). For fluorescent images, areas to image were identified based on DAPI (nuclei) or the epithelial marker, E-Cadherin (CDH1), to eliminate bias. Multiple images (2–3) were taken per prostate lobe for analysis from an *n* = 3–5 mice per treatment group representing 2–4 litters per treatment group. Final measures were averaged per tissue such that the animal was the *n* value. All *n* values are also reported in the respective figure legends. Quantification of images was performed by an experimenter blinded to treatment conditions. Prostate smooth muscle (ACTA2+) thickness was quantified using the Image J (v.1.46r) measure feature by taking multiple measures (at least 3) around each duct from each image. Cleaved caspase 3 (Casp3) and Ki67 positive cells were quantified using the counter feature in Image J. Casp3 positive cells were expressed as a percentage of total cells (DAPI+) within the image. Ki67-positive cells were counted in the prostate epithelium and stroma. These counts were then expressed as a percentage of all epithelia (CDH1+), all stroma (CDH1-, non-muscle), or total cells present within the image. Prostate collagen was visualized using picrosirius red staining as described previously [32]. Raw images were collected as RGB 8-bit images in two channels, one specific to picrosirius red and one nonspecific channel, images were analyzed using Image J. The raw, nonspecific image was subtracted from the picrosirius red image to avoid errors sourced from the autofluorescence of the prostate membrane. Regions of interest (ROI) were identified in the extracellular matrix where the plane of collagen was in focus and did not appear distorted by the tissue embedding and sectioning process. Then, the image was thresholded to convert the color image to a binary black and white to distinguish pixels with positive signals. The total number of positive pixels in the image ROIs was divided by the total number of pixels within the ROIs to provide the total percentage of collagen density within those regions. Each unique prostate lobe was analyzed using multiple images. H&E staining of bladder sections was performed as previously described [24]. Bladder smooth muscle thickness was quantified using the Image J (v.1.46r) measure feature. Bladder smooth muscle was measured in at least 4 regions from an entire bladder cross section from 1–2 slides and averaged such that one value was obtained for each tissue (*n* = 6–12 mice per treatment group, representing at least 5 litters per treatment group).

### 2.5. Creatinine Assays

Blood was collected via cardiac puncture after euthanasia, allowed to clot for 30 min, and centrifuged at 2000× *g* for at least 5 min. Serum was then used for serum creatinine assay per the manufacturer’s instructions (#80350, Crystal Chemistry, Elk Grove Village, IL, USA). Serum from littermates of the same sex was pooled if required for a sufficient sample. Free-catch urine was collected from mice when scruffed, days prior to euthanasia and analyzed using a urine creatinine assay per manufacturer’s instructions (#80350, Crystal Chemistry, Elk Grove Village, IL, USA). *n* = 4–5 mice per treatment group, representing 4–5 L per treatment group.

### 2.6. Statistics

Statistical analysis was conducted with GraphPad PRISM 6 (version 9.2). The data were tested for normality using Shapiro-Wilk and Kolmogorov-Smirnov tests. The variance was determined using Bartlett’s test or Brown-Forsythe’s test. If normality and variance tests were passed, one-way ANOVA was used to determine significance followed by Tukey’s multiple comparisons tests. If data had unequal variance Welch’s one-way ANOVA was used followed by Dunn’s multiple comparisons. If data failed to meet normality assumptions, data were analyzed using a Kruskal–Wallis test followed by Dunn’s multiple comparisons tests. A *p*-value of <0.05 was considered significant. All *n* values are also indicated in the figure legends along with specified statistical tests used.

## 3. Results

### 3.1. PCBs Decrease Ventral Prostate Wet Weight in Developmentally Exposed Male Mice

**Figure 1:**
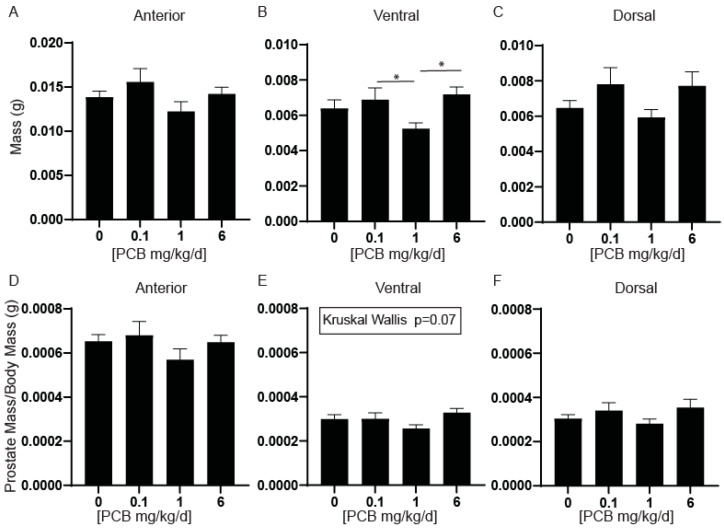
Developmental exposure to 1 mg/kg PCBs daily decreases ventral prostate wet weight in male offspring compared to other PCB treatment groups. Dams were exposed to PCBs orally through gestation and lactation. Prostates were collected and weighed from young adult offspring at 6 weeks of age. Quantification of average mass in (**A**) anterior, (**B**) ventral, and (**C**) dorsal prostates as well as mass normalized to body mass in (**D**) anterior, (**E**) ventral, and (**F**) dorsal prostates. Results are mean ± SEM, *n* = 15–18 per treatment group. *p* values ≤ 0.05 as determined by (**A**) Welch’s ANOVA test and (**B**–**F**) Kruskal–Wallis Test followed by Dunn’s multiple comparisons tests. * and bar indicate significance between dosing groups.

We previously observed that developmental PCB exposure can increase relative bladder volume in male mice at 4 weeks of age [24] and lead to voiding dysfunction in male and female mice at 6 weeks of age [12]. Increased growth of the prostate can impinge upon the urethra and contribute to enlarged bladder volumes and voiding dysfunction in rodent obstruction models [7,8]. Therefore, one goal of this study was to test whether developmental PCB exposure leads to changes in prostate growth or morphology in male offspring at 6 weeks of age. As a measure of growth, we first examined wet weights of the anterior, dorsal, and ventral prostate lobes. Ventral prostate lobe mass decreased in the 1 mg/kg PCB treatment group compared to the 0.1 and 6 mg/kg PCB treatment groups with no changes in any other prostate lobes (Figure 1A–C); however, when normalized to body mass, there were no significant differences in prostate lobe wet weights between treatment groups, though the same ventral prostate phenotype was trending toward significance with *p* = 0.07 (Figure 1D–F). Together these results indicate that prostate growth/obstruction is not likely contributing to voiding dysfunction in PCB-exposed male mice, but PCBs are capable of influencing raw ventral prostate wet weight.

**Figure 2:**
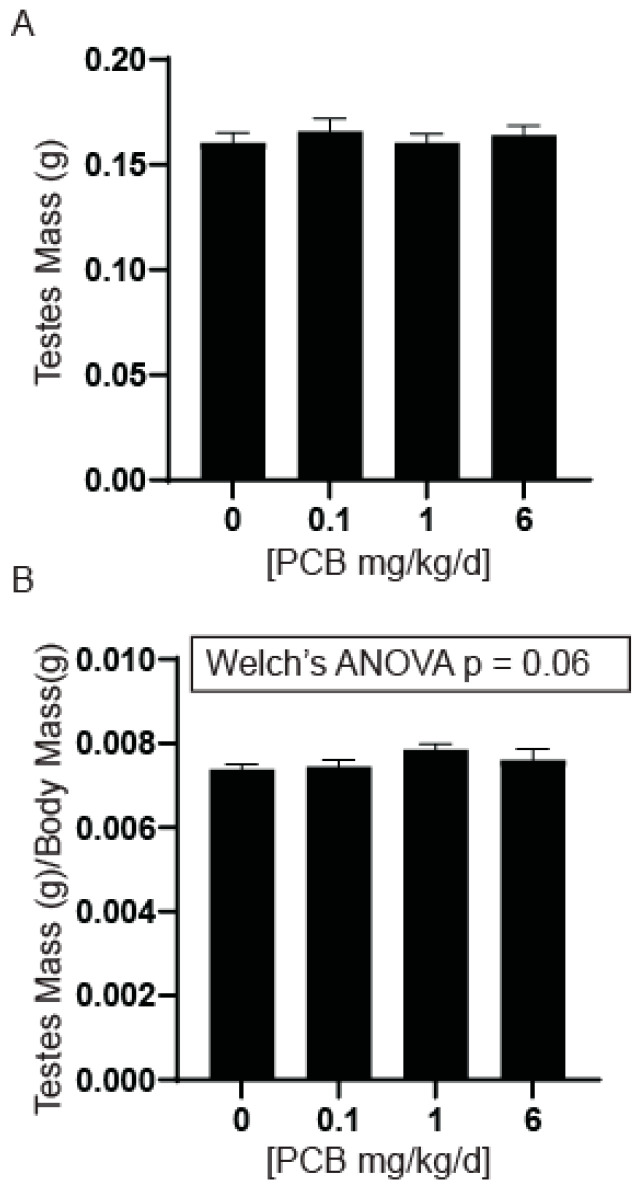
PCBs have no effect on testes mass in developmentally exposed male offspring. Dams were exposed to PCBs orally through gestation and lactation. Testes were collected from young adult male offspring at 6 weeks of age. Quantification of (**A**) average testes mass and (**B**) testes mass normalized to body mass. Results are mean ± SEM, *n* = 13–17 per treatment group. No significant difference was determined by the (**A**) Kruskal–Wallis test and (**B**) Welch’s ANOVA test.

Prostatic development, growth, and maturation are dependent upon testicular androgens [33,34,35,36]. Irregularities in testicular wet weight can indicate cellular toxicity or damage which can influence testosterone levels [37,38,39]. Since we observed a PCB-induced decrease in prostate wet weight and developmental exposure to PCBs has also been shown to reduce testes size in rats [40], we examined testes wet weight. There was no significant difference in testes wet weight between treatment groups (Figure 2A). When normalized to body mass there was a trend (*p* = 0.06) for an increase in testes/body mass in the 1 mg/kg PCB treatment group compared to control (Figure 2B).

### 3.2. PCBs Do Not Alter Prostate Smooth Muscle Thickness, Apoptotic Cells, or Proliferating Cells in Developmentally Exposed Male Mice

**Figure 3:**
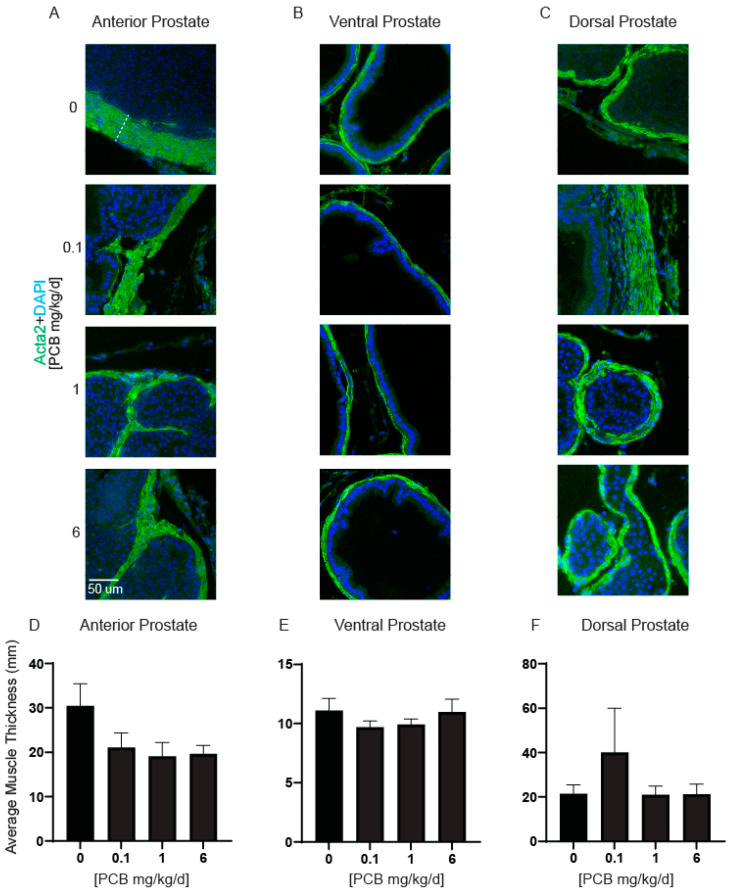
PCBs have no effect on prostate smooth muscle thickness in developmentally exposed male mice. Male mice were exposed to PCBs via maternal diet through gestation and lactation and prostates were collected at 6 weeks of age for immunohistochemistry. Representative images of (**A**) anterior, (**B**) ventral, and (**C**) dorsal prostates of each PCB treatment group incubated with antibodies targeting alpha-smooth muscle actin (Acta2, green) to label smooth muscle and DAPI (blue) to label nuclei. Quantification of average muscle thickness in (**D**) anterior, (**E**) ventral, and (**F**) dorsal prostates. Results are mean ± SEM, *n* = 3–5 prostates per treatment group. The dashed line in the first figure of panel A represents the thickness measure of Acta2 positive cells. No significant differences as determined by (**D**) Kruskal–Wallis test (**E**) one-way ANOVA and (**F**) Welch’s ANOVA test.

While the PCB mixture used here tends to decrease rather than promote prostate growth, it remains important to determine whether there are any PCB-induced changes to prostate morphology at the cellular level. Changes to prostate smooth muscle thickness, proliferation, and collagen density have all been reported in mouse models of LUTD with elevated bladder volumes or in conjunction with environmental chemicals such as 2,3,7,8-tetrochlorodibenzo-p-dioxin (TCDD, dioxin) [7,8,41]. We first analyzed prostate smooth muscle thickness (Figure 3A–C). Developmental PCB exposure did not cause any significant differences in prostate smooth muscle thickness in any prostate lobe (Figure 3D–F).

**Figure 4:**
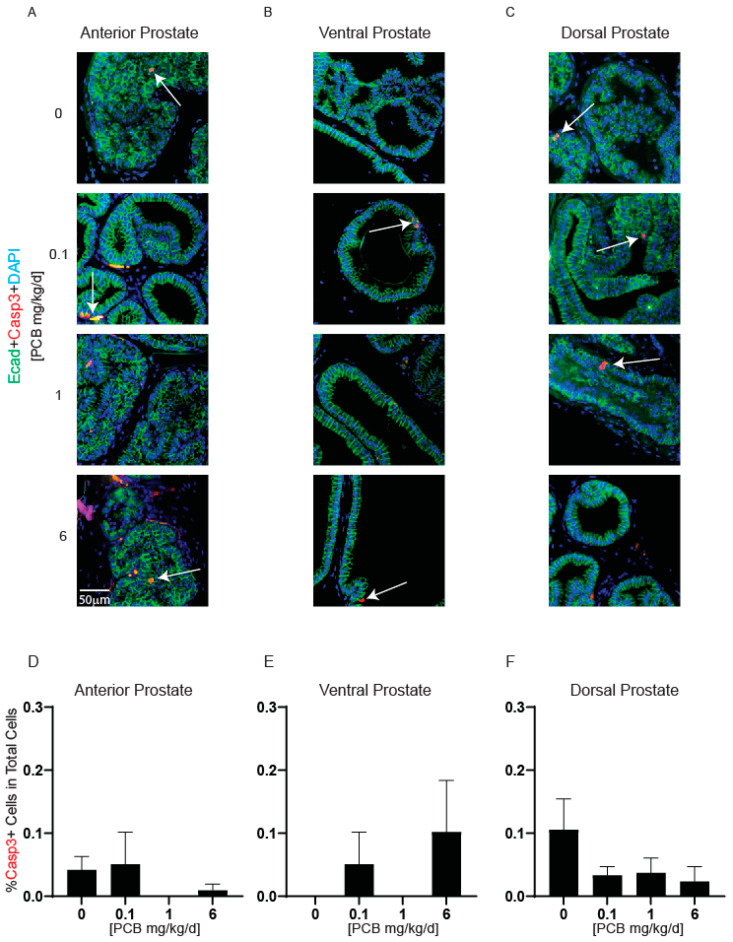
PCBs have no effect on prostate caspase 3 positive cells in developmentally exposed male mice. Male mice were exposed to PCBs via maternal diet through gestation and lactation and prostates were collected at 6 weeks of age for immunohistochemistry. Representative images of (**A**) anterior, (**B**) ventral, and (**C**) dorsal prostates of each PCB treatment group incubated with antibodies targeting cleaved caspase 3 (Casp3, red) to label apoptotic cells, e-cadherin (ECad) (CDH1, green) to label all epithelium and DAPI (blue) to label nuclei. Quantification of the percent of total cells Casp3 positive cells in (**D**) anterior, (**E**) ventral, and (**F**) dorsal prostate. Results are mean ± SEM, *n* = 3–5 per treatment group. No significant differences as determined by the Kruskal–Wallis test.

PCBs can cause apoptosis in other tissues [42,43], therefore we wanted to test whether PCBs altered apoptosis in the prostate. Cleaved caspase 3 was used as a marker of apoptotic cells. Few positive cells were detected in prostate tissues, and there were no significant differences among treatment groups (Figure 4A–F). This suggests that cell death does not contribute to PCB-induced differences in raw ventral prostate mass.

**Figure 5:**
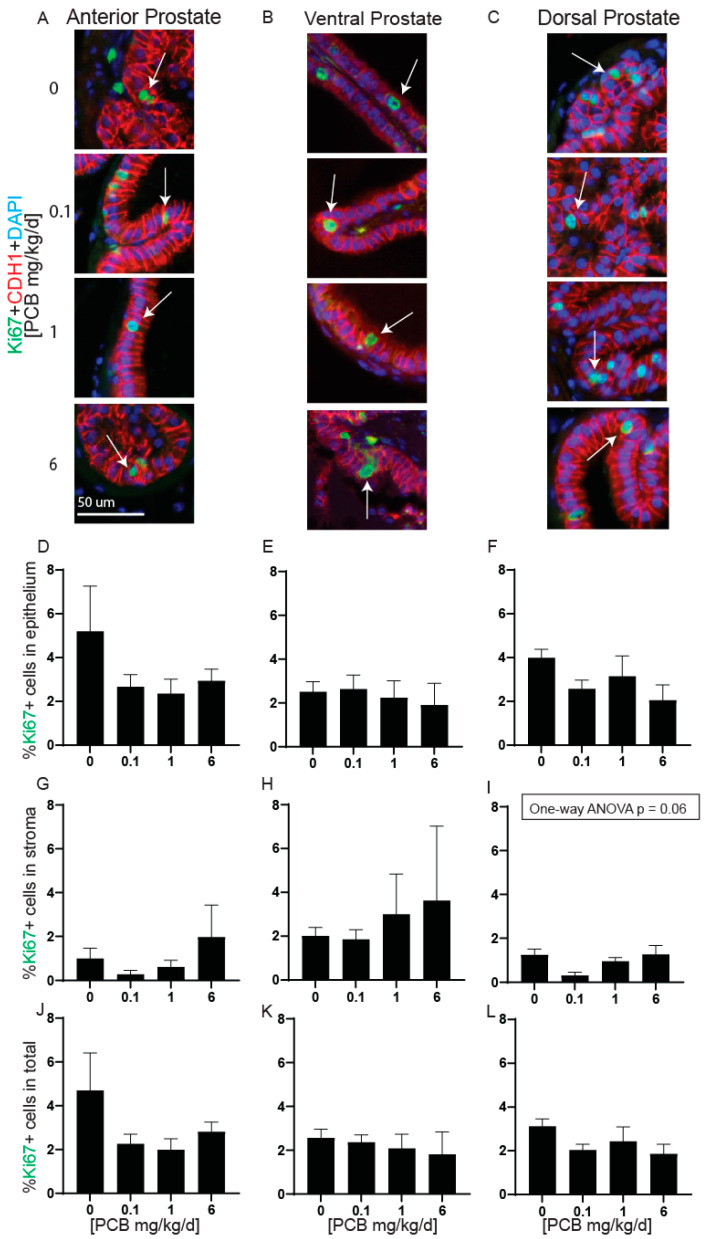
PCBs have no effect on prostate cell proliferation in developmentally exposed male mice. Mice were exposed to PCBs via the maternal diet throughout gestation and lactation, and prostates were collected from male offspring at 6 weeks of age for immunohistochemistry. Representative images of (**A**) anterior, (**B**) ventral, and (**C**) dorsal prostates at each PCB exposure group incubated with antibodies targeting Ki67 (green) to label replicating cells, e-cadherin (CDH1, red) to label epithelium and DAPI (blue) to label nuclei. Quantification of the percent Ki67+ cells in (**D**–**F**) epithelium, (**G**–**I**) stroma, and (**J**–**L**) total. Results are mean ± SEM, *n* = 3–5 prostates per group. No significant differences as determined by (**E**,**I**,**K**) one-way ANOVA, (**D**,**F**,**G**,**H**,**L**) Kruskal–Wallis test and (**J**) Welch’s ANOVA.

The proliferation of the prostate has been linked to altered prostate mass [8]. Therefore, we tested whether PCBs altered prostate cell proliferation. There were no significant differences in the proportion of total Ki67 positive cells or Ki67 positive cells within the epithelium or stroma independently in any prostate lobe (Figure 5D–L). However, there was a trend for decreased Ki67-positive cells in the dorsal prostate stroma in the 0.1 mg/kg/d PCB treatment group compared to vehicle control (Figure 5I).

### 3.3. PCBs Decrease Prostate Collagen Density in the Anterior Prostate in Developmentally Exposed Male Mice

**Figure 6:**
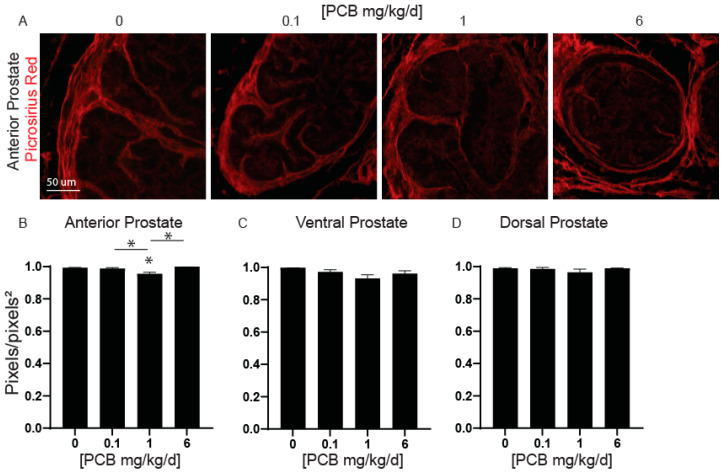
PCBs decrease collagen fiber density in the anterior prostate of developmentally exposed male mice. Dams were exposed to PCBs orally through gestation and lactation. Prostates were collected from young adult offspring at 6 weeks of age. Prostate sections were incubated with picrosirius red to visualize collagen (red). Representative images of (**A**) anterior prostate with each PCB exposure group. Quantification of collagen density (red, picrosirius red) in (**B**) anterior, (**C**) ventral, and (**D**) dorsal lobes of the prostate. Results are mean ± SEM, *n* = 3–4 per treatment group. *p* values ≤ 0.05 as determined by (**B**,**C**) one-way ANOVA followed by Tukey’s multiple comparison test, (**D**) Kruskal–Wallis test. * indicates a significant difference from control. * and bar indicate significance between dosing groups.

Changes in collagen fibril distribution and remodeling in the prostate and bladder extracellular matrix have been shown to occur in an aged mouse model developmentally exposed to dioxin [8]. We have recently shown that developmental PCB exposure can decrease bladder collagen density in young mouse offspring [25]. Therefore, we wanted to determine whether developmental exposure to PCBs can alter collagen density within the prostate in young adult mouse offspring and whether these changes coincide with observed changes in prostate wet weight (Figure 1) or previously reported voiding dysfunction [12]. Collagen density was significantly decreased in the anterior prostate of the 1 mg/kg PCB treatment group compared to vehicle control and compared to all other PCB treatment groups (Figure 6B). There were no significant changes in the ventral or dorsal prostate (Figure 6C,D). Thus, observable changes in anterior prostate collagen density occur in 6-week-old mice that were developmentally exposed to PCBs.

### 3.4. PCBs Increase Bladder Volume in Developmentally Exposed Mice

**Figure 7:**
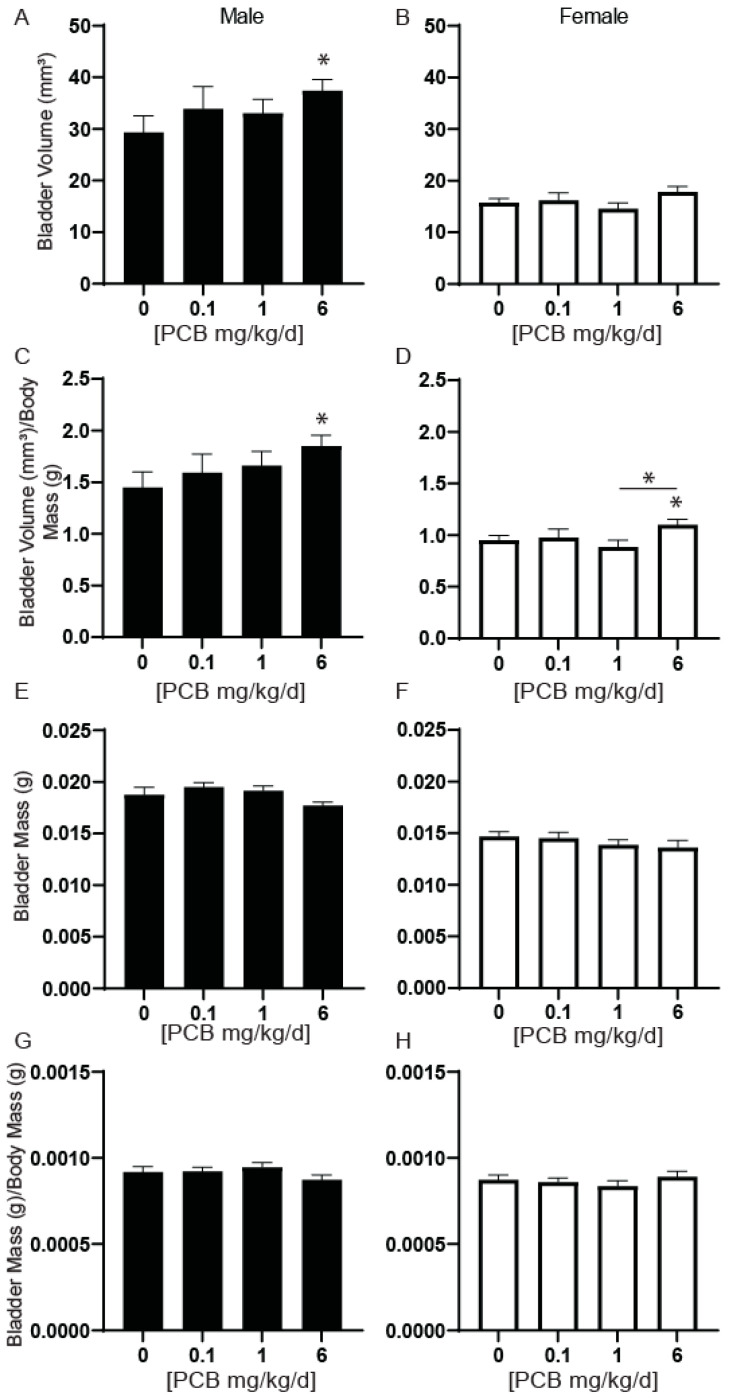
Developmental exposure to PCBs increases bladder volume. Dams were exposed to PCBs orally through gestation and lactation. Bladder volumes were collected from young adult offspring at 6 weeks of age. Quantification of bladder volume in (**A**) male and (**B**) female mice and bladder volumes normalized to body mass in (**C**) male and (**D**) female mice. Results are mean ± SEM, *n* = 18–28 per treatment group. Quantification of bladder mass in (**E**) male and (**F**) female mice and bladder mass normalized to body mass in (**G**) male and (**H**) female mice. Results are mean ± SEM, *n* = 8–21 per treatment group. *p* values ≤ 0.05 as determined by Kruskal–Wallis tests followed by Dunn’s multiple comparisons test. * indicate significance from the control and * and bar denotes significance between treatment groups.

Mouse models of prostate-induced voiding dysfunction or obstruction also typically display alterations to bladder volume [7,8]. We previously found that 4-week-old male mice developmentally exposed to the same MARBLES PCB mixture used here had increased relative bladder volume normalized to body mass at the 6 mg/kg PCB treatment group compared to vehicle control [24]. A second goal of our study was to determine whether this phenotype persisted in older animals. Relative bladder volume in mice at 6 weeks of age was significantly increased in male mice at the 6 mg/kg PCB treatment group compared to the vehicle control (Figure 7A). We separately assessed this endpoint in female mice as well. There were no differences in this parameter in female mice (Figure 7B). When relative bladder volume was normalized to body mass, a significant increase remained in male mice at the 6 mg/kg PCB treatment group compared to vehicle control (Figure 7C) and was also now present in female mice in the 6 mg/kg PCB treatment group compared to vehicle control and 1 mg/kg PCB treatment group (Figure 7D). Therefore, this PCB induced phenotype not only persisted to at least 6 weeks of age in male mice but was also now detected in female mice. These data also suggest that PCB-induced changes to bladder volume occur in the absence of changes in prostate mass in male mice.

**Figure 8:**
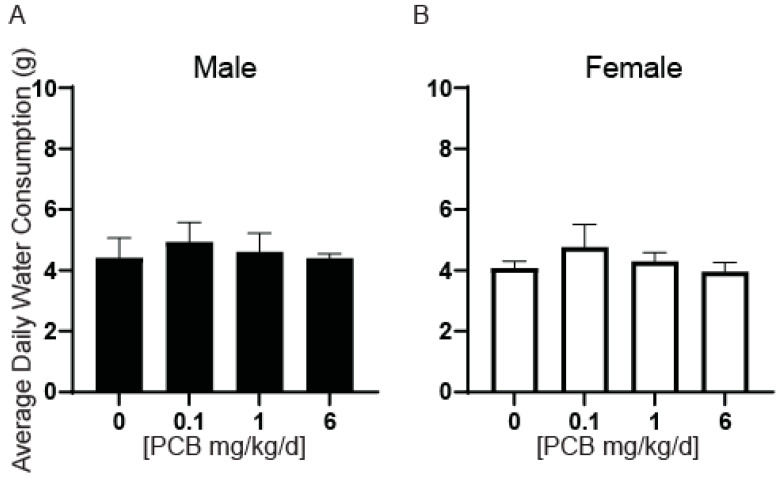
Developmental exposure to PCBs does not alter average daily water consumption. Dams were exposed to PCBs orally through gestation and lactation. Water consumption was measured for young adult offspring prior to the end of the study and tissue collection. Quantification of average daily water consumption in (**A**) male and (**B**) female mice. Results are mean ± SEM, *n* = 4–8 cages per treatment group. No significant differences as determined by (**A**) Kruskal–Wallis test and (**B**) one-way ANOVA.

**Figure 9:**
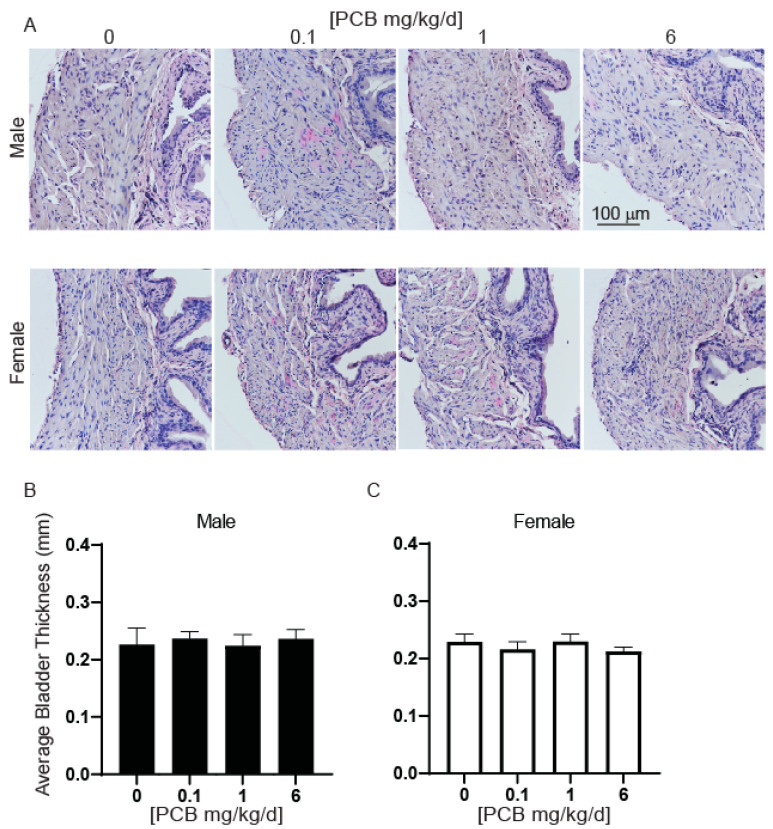
Developmental exposure to PCBs does not alter bladder thickness. Dams were exposed to PCBs orally through gestation and lactation. Bladders were collected from young adult offspring at 6 weeks of age. (**A**) Representative images of bladders in male and female mice for each treatment group. Quantification of average bladder thickness in (**B**) male and (**C**) female mice. Results are mean ± SEM, *n* = 6–12 per treatment group. No significant differences as determined by Kruskal–Wallis test.

We next determined whether changes in relative bladder volume had accompanying changes in bladder mass. There were no significant differences in bladder mass or bladder mass normalized to body mass in male or female mice (Figure 7E–H). Since changes in water consumption could contribute to bladder volumes, we also measured the daily consumption per animal per cage and found no significant differences (Figure 8). There were also no significant differences in bladder wall thickness (Figure 9A–C).

### 3.5. PCBs Increase Serum and Urine Creatinine Concentrations in Developmentally Exposed Male Mice

**Figure 10:**
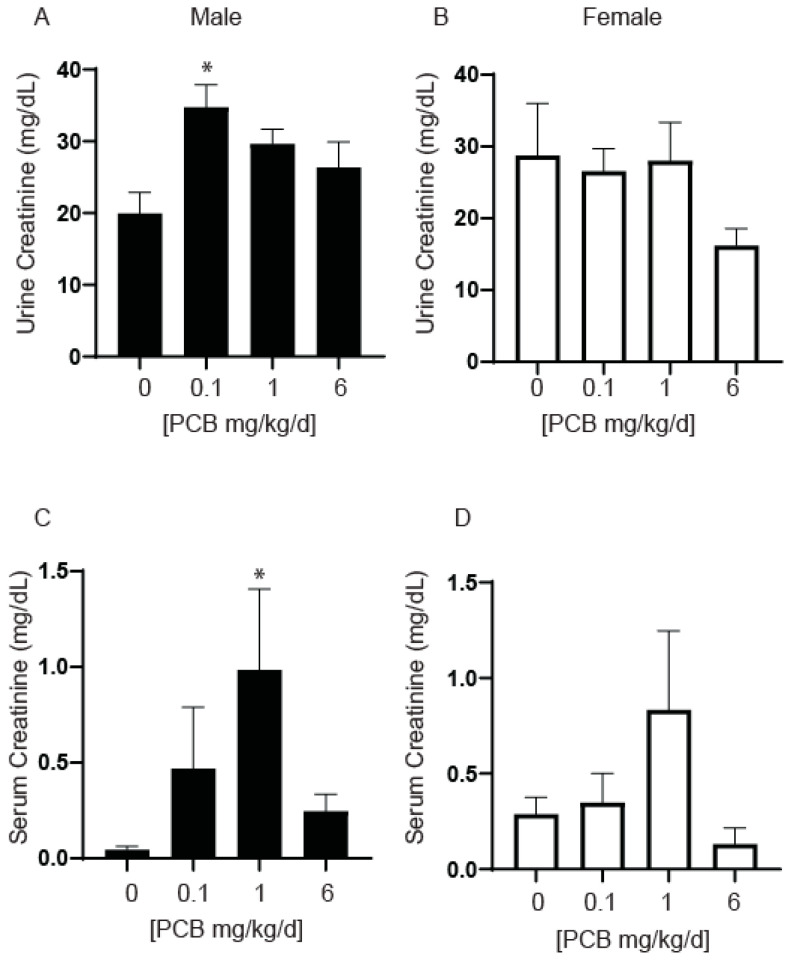
Developmental exposure to PCBs can increase serum and urine creatinine concentrations. Dams were exposed to PCBs orally through gestation and lactation. Quantification of average urine creatinine concentration in (**A**) male and (**B**) female mice, and average serum concentration in (**C**) male and (**D**) female mice at 6 weeks of age. Results are mean ± SEM, *n* = 4–5 per treatment group. *p* values ≤ 0.05 as determined by (**A**,**B**) one-way ANOVA followed by Tukey’s multiple comparisons and (**C**,**D**) Kruskal–Wallis Test followed by Dunn’s multiple comparisons. * indicates significant difference from the vehicle control.

To determine whether altered kidney function could contribute to observed bladder volume changes, we examined urine and serum creatinine concentrations. Urine creatinine concentrations were elevated in male mice in the 0.1 mg/kg PCB treatment group compared to vehicle control (Figure 10A). Serum creatinine concentrations were increased in male mice in the 1 mg/kg PCB treatment group compared to vehicle control (Figure 10C). There were no significant changes in urine or serum creatinine concentrations in female mice (Figure 10B,D). This could indicate either changes in kidney function, as kidneys should filter creatinine from the blood to the urine or changes in metabolic muscle breakdown leading to elevated creatinine in the body; however, the 6 mg/kg male and all female PCB treatment groups were unaffected and therefore do not overlap with observed bladder volume changes within these groups.

## 4. Discussion

Changes within the prostate especially in relation to volume and/or extracellular matrix composition are associated with urinary voiding dysfunction in men and animal models. A previous study observed developmental PCB exposure using the same mixture used here, lead to smaller voids, more drop-like voiding behavior, and increased normalized peak pressure in male mice [12]. This voiding phenotype is also observed in mouse models of prostate growth-induced obstruction [7,8]. Here we sought to test whether developmental exposure to the MARBLES PCB mixture altered prostate morphology in young adult mouse offspring which could provide one mechanism driving PCB-induced voiding phenotypes in male mice. We observed that PCBs do not increase prostate mass. Therefore, it is unlikely that prostate growth and obstruction contribute to PCB-induced voiding phenotypes observed in male mice [12]. However, this does not preclude the possibility that PCB-induced changes to the prostate account for some of the sex and dose-dependent differences observed in voiding phenotypes in Kennedy et al., 2022 [12]; it just suggests that they occur through other mechanisms besides obstruction related to prostate growth. For example, PCB-induced changes in the prostate were only observed at one PCB dose and included a decrease in ventral prostate wet weight at the 1 mg/kg PCB treatment group vs. other PCB treatment groups, and a decrease in collagen density in the anterior prostate at the 1 mg/kg PCB treatment group compared to control and all other PCB treatment groups. Animals in this same 1 mg/kg PCB treatment group had the fewest number of changes to male mouse voiding function in comparison to the 0.1 or 6 mg/kg PCB treatment groups [12], with no differences in the size of urine spots in this group vs. control. Therefore, we cannot rule out the possibility that PCB-induced changes to the prostate influence the voiding physiology in male mice, especially at the 1 mg/kg PCB treatment [12]. Interestingly, we did find several other intriguing results in that PCBs can alter prostate morphology–specifically collagen density-suggesting that collagen fiber distribution or remodeling may occur following developmental PCB exposures. The implications for these prostate-dependent changes in models of inflammation or aging are intriguing areas of future study.

Developmental exposure to PCBs in mice also resulted in increased relative bladder volume normalized to body mass. This was previously observed at 4 weeks of age in developmentally exposed male mice [24], whereas here we demonstrated that this phenotype persists to at least 6 weeks of age. Interestingly, at 6 weeks of age, there was also an increase in relative bladder volume among the female mice in the 6 mg/kg PCB treatment group, which was not observed at 4 weeks of age. These results indicate that at this timepoint changes continue to occur and manifest in developmentally exposed offspring- 3 weeks after the offspring’s last exposure to PCBs. Changes in relative bladder volume are not accompanied by changes in water consumption, bladder thickness, bladder mass or increases in prostate mass. This suggests that extensive bladder remodeling such as seen with bladder compensation and eventual decompensation in severe models of obstruction [8] is not likely to occur. Serum and urine creatinine levels did differ with PCB exposure in male mice but at different concentrations than bladder volume changes. This could indicate changes in kidney function, as the kidneys are responsible for filtering creatinine into the urine, or in changes to metabolic breakdown of muscle to produce higher levels of creatinine in the body. However, the fact that these changes were not observed in the highest PCB treatment group or at all in female mice suggests they are not likely driving PCB-induced increases in relative bladder volume at the 6 mg/kg PCB treatment group. Nevertheless, the consequences of these changes on voiding function are an area of future study. While we cannot determine the exact mechanism driving PCB-induced increases in relative bladder volume in this model, our results help to rule out one of the possible mechanisms- prostatic enlargement leading to obstruction–since we found that prostate growth does not occur following developmental PCB exposure in our model. Several other possibilities exist including, urethral dysfunction, bladder detrusor sphincter dyssynergia, or changes to the nervous system such that pressure/volume information is not adequately relayed to induce voiding. These possibilities could also contribute to the increased bladder volume and voiding dysfunction phenotypes observed in female mice. The exact cause of this phenotype is an ongoing area of future study along with mechanisms underlying sex differences. Expanding the timeline of assessing LUT morphology and function will help determine the trajectory of these changes and whether sex differences can be attributed to differences in developmental maturity between male and female mice.

Our data on developmental PCB exposure effects on the prostate adds to the growing body of literature on persistent environmental toxicants and their impacts on the lower urinary tract. Developmental exposure to the contaminant TCDD (dioxin) can exacerbate urinary dysfunction in mice treated with testosterone and estradiol to mimic hormonal changes of aging men [10,11] and can predispose genetically prone male mice to prostatic disease [8]. It has been demonstrated that dioxin exposure can increase the number of Ki67-positive proliferating cells in the epithelium of the ventral prostate and increase smooth muscle thickness in the dorsal prostate [8,11]. The effects of TCDD (dioxin) are intriguing since a subset of PCBs have a similar structure to TCDD, and act through the same pathways. The MARBLES PCB mixture used in this study contains only one dioxin-like PCB, PCB118. However, it is unlikely that PCB118 is driving PCB-induced changes in the prostate and bladder since we observed no changes in proliferation or smooth muscle thickness in any of the lobes of the prostate. Examining a greater number of animals at this time point may be necessary to find very subtle changes in these parameters, but it is also likely that PCB118′s effects are either negligible or overshadowed by other more abundant congeners in the MARBLES PCB mixture. The MARBLES PCB mixture has demonstrated non-dioxin-like PCB activity in that it has been shown to bind ryanodine receptors [23]. Determining the contribution of each dioxin-like and non-dioxin-like PCB on LUT function is an area of future study. Nevertheless, these findings are important as they demonstrate that environmental contaminants like TCDD (dioxin) and PCBs can alter voiding function well into adulthood and can impact prostate growth and morphology in several different ways [10,11,12].

PCB-induced decreases in prostate mass reported here are consistent with other studies that used different combinations of PCBs [44]. For example, a dose of 2 mg/kg/d of PCB mixture Aroclor 1254 via intraperitoneal injection for 30 days decreased ventral prostate weight in rats [45]. These same rats had decreased triiodothyronine (T3), thyroxine (T4), testosterone, and estradiol, as well as increased thyroid-stimulating hormone (TSH) [45]. PCB-exposed rats also had a significant decline in androgen and estrogen receptors in the ventral prostate [45], suggesting thyroid and steroid receptor signaling pathways as mechanisms leading to decreased prostate mass. Despite the similar prostate phenotype, there are several differences between other parameters in our dosing paradigm and the one used with rats [45], suggesting different mechanisms may underlie decreased prostate mass phenotypes depending on the PCB congeners used. First, the mice exposed in the current study did not have any changes in testicular mass, indicating that there were no overt signs of testosterone dysregulation as was seen in the Aroclor rat study [45]. Second, P28 male mice exposed to the same MARBLES PCB mixture and dosing paradigm as this study, had an increase in T3 levels at the 0.1 mg/kg PCB treatment group when compared to the vehicle control [27]. This is the opposite effect seen in the Aroclor-dosed rat study (decreased T3) [45]. However, future study is necessary to assess serum testosterone levels and expression of androgen and estrogen receptors to determine the role of this pathway in this model. Changes in oxidative stress could also play a role in the observed phenotypes. Antioxidant enzymes were decreased in the rat study, suggesting possible oxidative stress [45,46]. Whether developmental exposure to PCBs in mice induces oxidative stress leading to decreased ventral prostate weight is unknown and is an area of future study.

Collagen formation and remodeling may be common targets of environmental exposures. In one study, exposure to TCDD during development led to a change in the distribution of collagen fiber sizes when adult mice were then also challenged with a combination of estradiol and testosterone to mimic the aging process [8]. In this study, we observed that developmental PCB exposure at the 1 mg/kg treatment group decreased anterior prostate collagen density in 6-week-old mouse offspring. Additionally, we have shown PCBs can lead to decreased collagen density in the bladder of developmentally exposed mice but only at the 0.1 mg/kg treatment group and predominantly in female mice [25]. Together this adds to the growing body of evidence that dioxin, as well as PCBs, can interfere with the extracellular matrix in the bladder and the prostate in a dose and sex-dependent manner. The mechanisms underlying these changes are active areas of research. Prostate collagen density can be altered by several mechanisms, including inflammation. Inflammation in the prostate increases collagen density [47,48,49] and can lead to more frequent voiding patterns [49]. However, in the current study, we observed a decrease in collagen density which would not be expected of an immune-mediated inflammatory response; however, there are other routes that could alter collagen density and remodeling. The secretion of immune mediators such as IL-1 has been shown to decrease collagen fiber formation [50]. Whether inflammation is linked to the observed phenotype in collagen density here and whether this is mediated through cytokines such as IL-1 is an area of future study. Another mechanism that can influence collagen density could involve the recruitment or mechanical properties of collagen fibers [51]. Examining these endpoints in our developmental PCB exposure model and determining whether mechanical distensibility is altered in the bladder and/or prostate is an area of future study.

This research lays the foundation for future studies and raises the intriguing hypothesis as to whether PCB levels in humans can be correlated with prostate size and/or collagen density. This is of considerable interest as both of these parameters are linked to lower urinary tract symptoms in patients. Examining benign prostate or LUT symptoms in exposure cohorts would greatly add to our understanding of the effects of PCBs on the lower urinary tract. Environmental toxins are of interest because, unlike genetic factors, they are in part modifiable risk factors whose levels could be modified by changes to lifestyle and exposures.

## Figures and Tables

**Table 1:** Antibodies used in this study.

**Primary Antibodies**	**Source**	**Company**	**Catalog #**	**Dilution**	
E-Cadherin (CDH1)	Rabbit	Cell Signaling Technology	3195s	1:250	
E-Cadherin (CDH1)	Mouse	BD Transduction Labs (via Fisher)	610181	1:250	
Cleaved caspase 3 (Casp3)	Rabbit	Cell Signaling Technology	9661S	1:200	
Ki67	Rabbit	Abcam	ab15580	1:200	
Smooth Muscle Actin (Acta2)	Goat	Fisher Scientific	PA518292	1:250	
**Secondary Antibodies**					**Pairing**
Anti-Mouse Alexa Fluor 594	Donkey	Jackson ImmunoResearch	715-545-150	1:250	CDH1
Anti-Rabbit Alexa Fluor 488	Donkey	Jackson ImmunoResearch	711-545-152	1:250	CDH1, Ki67
Anti-Rabbit Alexa Fluor 594	Goat	Jackson ImmunoResearch	111-585-144	1:250	Casp3
Anti-Goat Alexa Fluor 488	Donkey	Jackson ImmunoResearch	705-545-003	1:250	Acta2

#; number.

## Data Availability

Available upon request to corresponding author.
